# Supplementary material for: Theoretical and empirical quantification of the accuracy of polygenic scores in ancestry divergent populations
Source: Nat Commun. 2020 Jul 31;11:3865. doi: 10.1038/s41467-020-17719-y (PMC7395791; doi:10.1038/s41467-020-17719-y)
Supplement: Supplementary file 1 — Supplementary Information [file 41467_2020_17719_MOESM1_ESM.pdf]

Theoretical and empirical quantification of the accuracy of polygenic scores in ancestry divergent populations

Wang Y., Guo J., Ni G., Yang J., Visscher P.M. & Yengo L.

Supplementary Notes

1. Theoretical expectation of trans-ancestry accuracy of polygenic scores

We assume causal variants to be shared between ancestries but allow their effect sizes to vary from one ancestry to another. Therefore, ancestry-specific causal variants are a special case with non-zero effect sizes in only one ancestry. We also assume that effect sizes are correlated between population and denote ρ_b that correlation. We finally assume that the trait is controlled by M_C causal variants and that prediction is based on M_T independent genome-wide significant (GWS) SNPs.

We can write the phenotypic value of individual from population l as

$$(1) \qquad y_l = \sum_{j=1}^{M_C} \beta_{jl} \left(\frac{x_{jl}^{(c)} - 2p_{jl}^{(c)}}{\sqrt{2p_{jl}^{(c)}\left(1-p_{jl}^{(c)}\right)}} \right) + e_l,$$

where $x_{jl}^{(c)}$ is the minor allele count (MAC) at the jth causal variant in population l, β_{jl} is the effect size per standardized genotype at the jth causal variant in population l, $p_{jl}^{(c)}$ is the minor allele frequency (MAF) at the jth causal variant in population l, and e_l is a residual term measuring non-genetic effects in population l.

We hereafter denote $h_{jl}^{(c)} = 2p_{jl}^{(c)}\left(1 - p_{jl}^{(c)}\right)$.

We now consider that the discovery GWAS is performed in individuals of Population 1 and accuracy is evaluated in individuals of Population 2. We denote $\hat{y}_l$ as the PGS calculated in individuals of population l. The squared correlation R_2^2 between y_2 and $\hat{y}_2$can be expressed as

$$(2) \qquad R_2^2 = \frac{\text{cov}^2(y_2,\hat{y}_2)}{\text{var}(\hat{y}_2)\text{var}(y_2)} = \left[\frac{\text{cov}(y_2,\hat{y}_2)}{\text{cov}(y_1,\hat{y}_1)}\right]^2 \times \frac{\text{cov}^2(y_1,\hat{y}_1)}{\text{var}(\hat{y}_1)\text{var}(y_1)} \times \frac{\text{var}(\hat{y}_1)}{\text{var}(\hat{y}_2)} \times \frac{\text{var}(y_1)}{\text{var}(y_2)}.$$

We assume, without loss of generality, that $\text{var}(y_1) = \text{var}(y_2) = 1$. Therefore, Equation (2) can be rewritten as

$$(3) \qquad R_2^2 = R_1^2 \times \left[\frac{\text{cov}(y_2,\hat{y}_2)}{\text{cov}(y_1,\hat{y}_1)}\right]^2 \times \frac{\text{var}(\hat{y}_1)}{\text{var}(\hat{y}_2)},$$

where R_1^2 denotes the prediction accuracy in individuals of same ancestry as in the discovery sample, i.e. Population 1.

Equation (3) depends on two parameters, which can be evaluated without observing the phenotype in the target population. Those parameters are R_1^2 which can be obtained from previous prediction studies in individuals of Population 1; and $\text{var}(\hat{y}_1)/\text{var}(\hat{y}_2)$, which can be directly

estimated in Population 2 or evaluated using data from a reference panel. The only unspecified parameter is $\text{cov}(y_2, \hat{y}_2)/\text{cov}(y_1, \hat{y}_1)$ for which we derive below an approximation.

We assume causal SNP effect sizes to be normally distributed, i.e.

(4) $$\begin{bmatrix}\beta_{j1}\\ \beta_{j2}\end{bmatrix} \sim \mathcal{N}\left(\begin{bmatrix}0\\0\end{bmatrix}, \frac{1}{M_C}\begin{bmatrix} h_1^2 & \rho_b\sqrt{h_1^2 h_2^2} \\ \rho_b\sqrt{h_1^2 h_2^2} & h_2^2\end{bmatrix}\right),$$

where h_l^2 denotes the heritability in population l.

Under the assumption that effect sizes of causal SNPs are independent random variables, we have that $\mathbb{E}[\beta_{il}\beta_{\text{j}l}] = 0$ when $i \neq j$, and given that $\mathbb{E}[\beta_{j1}\beta_{j2}] = \rho_b\sqrt{h_1^2 h_2^2}/M_C$ we can write

(5) $$\frac{\text{cov}(y_2,\hat{y}_2)}{\text{cov}(y_1,\hat{y}_1)} \approx \rho_b \times \sqrt{\frac{h_2^2}{h_1^2}} \times \frac{\sum_{k=1}^{M_T}\sqrt{\frac{h_{k2}^{(t)}}{h_{k1}^{(t)}}}\left(\sum_{j=1}^{M_C} r_{jk,1} r_{jk,2}\right)}{\sum_{k=1}^{M_T}\left(\sum_{j=1}^{M_C} r_{jk,1}^2\right)},$$

where $r_{jk,l} = cov\left(x_{kl}^{(t)}, x_{jl}^{(c)}\right)/\sqrt{h_{jl}^{(c)} \times h_{kl}^{(t)}}$ is the LD correlation between causal SNP j and GWS SNP k in population l and $h_{kl}^{(t)} = var(x_{kl}) = 2p_{kl}^{(t)}\left(1 - p_{kl}^{(t)}\right)$. Hence, we get Equation (1) in the main text.

To illustrate the above approximation, we first derive the ordinary least squared (OLS) estimator $\hat{\beta}_{k1}^{(\text{t})}$ (OLS estimator of SNP k calculated in individuals of Population 1) conditional on the β_{j1}'s is such that

(6) $$\text{E}[\hat{\beta}_{k1}^{(t)}] = \frac{\text{cov}(x_{k1},y_1)}{\text{var}(x_{k1})} = \sum_{j=1}^{M_C}\frac{\beta_{j1}}{\sqrt{h_{j1}^{(c)}}} \times \frac{\text{cov}(x_{k1}^{(t)},x_{j1}^{(c)})}{\text{var}(x_{k1}^{(t)})} = \frac{1}{\sqrt{h_{k1}^{(t)}}}\sum_{j=1}^{M_C}\beta_{j1} r_{jk,1}.$$

We can also express the variance of $\hat{\beta}_{\text{k}1}^{(\text{t})}$ as

(7) $$\text{var}(\hat{\beta}_{k1}^{(t)}) = \frac{\text{var}(y_1) - \text{E}[\hat{\beta}_{k1}^{(t)}]^2\text{var}(x_{k1}^{(t)})}{N_1\text{var}(x_{k1}^{(t)})} = \frac{\text{var}(y_1) - \text{E}[\hat{\beta}_{k1}^{(t)}]^2 h_{k1}^{(t)}}{N_1 h_{k1}^{(t)}} = \frac{\text{var}(y_1)}{N_1 h_{k1}^{(t)}} - \frac{1}{N_1}\text{E}[\hat{\beta}_{k1}^{(t)}]^2.$$

In the target sample of Population 2, $\hat{y}_2$ is defined as

(8) $$\hat{y}_2 = \sum_{k=1}^{M_T}\hat{\beta}_{k1}^{(t)} x_{k2}^{(t)} = \mu_{\text{PGS}} + \sum_{k=1}^{M_T}\hat{\beta}_{k1}^{(t)}\left[x_{k2}^{(t)} - 2p_{k2}^{(t)}\right],$$

where $\mu_{\text{PGS}} = \sum_{k=1}^{M_T}\hat{\beta}_{k1}^{(t)} 2p_{k2}^{(t)}$.

We note that μ_{PGS} is constant for all individuals in the target sample. Therefore, conditional on SNP true effect sizes $\boldsymbol{\beta} = \left((\beta_{11}, \beta_{12}), \dots, (\beta_{j1}, \beta_{i2}), \dots, (\beta_{M_C1}, \beta_{M_C2})\right)$, the

(9) $\mathrm{cov}(\hat{y}_2, y_2|\,\boldsymbol{\beta}) = \mathrm{cov}\left(\sum_{k=1}^{M_T} \hat{\beta}_{k1}^{(t)}\left[x_{k2}^{(t)} - 2p_{k2}^{(t)}\right], y_2\right).$

$$= \mathrm{cov}\left[\sum_{k=1}^{M_T} \hat{\beta}_{k1}^{(t)}[x_{k2}^{(t)} - 2p_{k2}^{(t)}], \sum_{j=1}^{M_C} \beta_{j2}\left(\frac{x_{j2}^{(c)} - 2p_{j2}^{(c)}}{\sqrt{h_{j2}^{(c)}}}\right) + e_2\right]$$

$$= \sum_{k=1}^{M_T} \sum_{j=1}^{M_C} \mathrm{E}[\hat{\beta}_{k1}^{(t)}]\beta_{j2}\sqrt{h_{k2}^{(t)}} \times \frac{\mathrm{cov}(x_{k2}^{(t)}, x_{j2}^{(c)})}{\sqrt{h_{j2}^{(c)} h_{k2}^{(t)}}}$$

$$= \sum_{k=1}^{M_T} \sum_{j=1}^{M_C} \sqrt{\frac{h_{k2}^{(t)}}{h_{k1}^{(t)}}} \times \beta_{j2} r_{jk,2}\left(\sum_{i=1}^{M_C} \beta_{i1} r_{ik,1}\right)$$

$$= \sum_{k=1}^{M_T} \sqrt{\frac{h_{k2}^{(t)}}{h_{k1}^{(t)}}}\left(\sum_{j=1}^{M_C} \beta_{j1}\beta_{j2} r_{jk,1} r_{jk,2} + \sum_{i\neq j} \beta_{i1}\beta_{j2} r_{ik,1} r_{jk,2}\right)$$

Similarly, we can show that

(10) $\mathrm{cov}(\hat{y}_1, y_1|\,\boldsymbol{\beta}) = \sum_{k=1}^{M_T}\left(\sum_{j=1}^{M_C} \beta_{j1}^2 r_{jk,1}^2 + \sum_{i\neq j} \beta_{i1}\beta_{j2} r_{ik,1} r_{jk,2}\right).$

We finally, approximate $\mathrm{cov}(y_2, \hat{y}_2)/\mathrm{cov}(y_1, \hat{y}_1)$ with its expectation as

(11) $\frac{\mathrm{cov}(y_2, \hat{y}_2)}{\mathrm{cov}(y_1, \hat{y}_1)} \approx \mathbb{E}\left(\frac{\mathrm{cov}(y_2, \hat{y}_2)}{\mathrm{cov}(y_1, \hat{y}_1)}\right) \approx \frac{\mathbb{E}(\mathbb{E}[\mathrm{cov}(\hat{y}_2, y_2|\boldsymbol{\beta})])}{\mathbb{E}(\mathbb{E}[\mathrm{cov}(\hat{y}_1, y_1|\boldsymbol{\beta})])}.$

2. Wright's F_{ST} calculation

The pairwise Wright's F_{ST}[1] between discovery and target dataset was used to estimate the genetic distance. For the sake of computational efficiency, we randomly sampled 50,000 individuals from the discovery dataset as the reference and calculated the F_{ST} using the software PLINK1.90[2] based on HapMap3 SNPs.

3. Estimates of allele frequency and LD score between ancestries

To further explore the impact of imputation on the estimates of LD correlation and MAF in our theory, we calculated the LD scores and MAF based on HapMap3 SNPs using both 1KGP WGS and UKB imputed data as the reference panel. The LD score in this study was calculated as 1 + $\overline{r^2} \times m$, where $\overline{r^2}$ is the mean LD r^2 between the target SNP and all other SNPs in the window and m is the number of SNPs in the window. In each ancestry, we calculated the LD score within 100 kb window using the software GCTA[3]. The 100 kb window was consistent with the arbitrary choice we made in the estimation of LD correlation in our theory. We used the heterozygosity calculated as $2p_{ij}(1-p_{ij})$, with p being the MAF of the ith SNPs in the jth population, to estimate the allele frequency correlation between ancestries.

4. GWASs using different sample sizes in the simulations

Since there is a general trend of growing sample sizes of GWASs for complex traits and diseases, such impact on the prediction performance in ancestry divergent populations remains to be explored. We focused on two simulation scenarios, where scenario 1 with M_C = 1,000 and h^2 = 0.5, and scenario 2 with M_C = 10,000 and h^2 = 0.25. We ran GWASs on different sample sizes using the genotype data of UK Biobank by randomly sampling the number of individuals ranging from 100,000 (100K) to 300,000 (300K) with an increment of 40,000 (40K). The GWAS was running using the simple linear regression association tests in PLINK1.90[2]. Both empirical and deterministic prediction accuracies (R^2) as well as relative accuracies (RA) were calculated in the same target populations as described in Methods.

5. GWASs for 5 quantitative traits and 3 common diseases in the UKB

We ran GWASs for 5 quantitative traits including body mass index (BMI), standing height (Height), low-density lipoprotein cholesterol (LDL), high-density lipoprotein cholesterol (HDL) and triglycerides (TG), and 3 diseases including asthma, type-2 diabetes (T2D) and hypertension (HTN). The diseases were defined using ICD-10 codes as mentioned in Zhu et al.[4]. The phenotypes were pre-adjusted by age, sex, recruitment centre, genotyping batches and the first 10 PCs. Noting for the quantitative traits, we firstly removed those outliers that were 3SD (standard deviation) away from the mean values. The residuals of them were then inverse-normal transformed with the aim to make them more normally distributed. Simple linear association tests were performed in 313,284 British using PLINK1.90[2].

6. Predictive performance using sub-significant SNPs

We also evaluated the predictive performance of PGS for traits and diseases in UK Biobank using sub-significant SNPs. We used a range of p-value thresholds to select PGS SNPs, i.e. 5×10^{-7}, 5×10^{-6}, 5×10^{-5}, 5×10^{-4}, 5×10^{-3} , 5×10^{-2} , 5×10^{-1} and 1. The process was the same as described in Methods to select the GWS SNPs. After selecting the PGS SNPs, we generated corresponding PGS and then calculated the prediction R^2 (see Methods) in the target populations.

7. Standard errors for the observed relative accuracies

We derive standard errors for the observed relative accuracies $\hat{R}_2^2/\hat{R}_1^2$ using the Delta-method based on Taylor series expansions. We first recall the asymptotic variance of the Pearson correlation coefficient $\hat{r}$, estimated in sample of N individuals:

(12) $\mathrm{var}(\hat{r}) \approx \frac{1-\hat{r}^2}{N}$.

Then, using the Delta-method, we can show that

(13) $\mathrm{var}(\hat{r}^2) \approx 4\hat{r}^2(1-\hat{r}^2)/N$.

Noting that the squared derivative of the function $f(x) = x^2$, is $[f'(x)]^2 = 4x^2$. Moreover, the variance of ratios lemma[5] posits that

$$\text{(14)} \quad \text{var}(u/v) \approx \left[\frac{\mathbb{E}(u)}{\mathbb{E}(v)}\right]^2 \left(\frac{\text{var}(u)}{[\mathbb{E}^2(u)]} - \frac{2\text{cov}(u,v)}{[\mathbb{E}(u)\mathbb{E}(v)]} + \frac{\text{var}(v)}{[\mathbb{E}^2(v)]}\right)$$

Therefore, assuming $\mathbb{E}(u) \approx \hat{u}$, and given that estimated accuracies $\hat{R}_1^2$ and $\hat{R}_2^2$ are independent (i.e. $\text{cov}(\hat{R}_1^2, \hat{R}_2^2) = 0$), we can finally write

$$\text{(15)} \quad \text{var}(\hat{R}_2^2/\hat{R}_1^2) \approx \left[\frac{\hat{R}_2^2}{\hat{R}_1^2}\right]^2 \left(\frac{4(1-\hat{R}_1^2)}{N_1\hat{R}_1^2} + \frac{4(1-\hat{R}_2^2)}{N_2\hat{R}_2^2}\right)$$

We can further extend these calculations to approximate the variance of the ratio (R_{LOA}) of the predicted loss of accuracy (1-RA_{pred}) over the observed loss of accuracy defined as 1-RA_{obs} (Supplementary Figure 12). Assuming RA_{pred} to be fixed (i.e. not random) then variance of R_{LOA} expressed as

$$\text{(16)} \quad \text{var}(R_{\text{LOA}})=\text{var}\left[\left(\frac{1-\text{RA}_{\text{pred}}}{1-\text{RA}_{\text{obs}}}\right)\right] \approx (1-\text{RA}_{\text{pred}})^2 \times \text{var}\left[\frac{1}{1-\text{RA}_{\text{obs}}}\right] \approx \text{var}(\text{RA}_{\text{obs}}) \times \frac{(1-\text{RA}_{\text{pred}})^2}{(1-\text{RA}_{\text{obs}})^4}.$$

Finally, we estimated the variance of this proportion across traits (denoted below as $\bar{R}_{LOA}$) using a leave-one-trait-out jackknife estimator defined as

$$\text{(17)} \quad \text{var}[\bar{R}_{LOA}] = \sqrt{(1-1/n_T)\sum_{k=1}^{n_T}(\bar{R}_{LOA} - R_{LOA,k})^2}$$

where $R_{LOA,k}$ is predicted proportion of the LOA explained by MAF and LD in trait k and n_T is the number of traits for which there is a significantly reduced prediction accuracy.

8. LDpred and SBayesR in the simulations

Numerous studies have shown that genome-wide methods such as LDpred[6] and SBayesR[7] taking advantage of LD information and GWAS summary statistics could overperform traditional LD clumping within European ancestry. We explored their performances in ancestry divergent populations under two simulation scenarios: Scenario 1 with M_C= 1,000 and h^2= 0.5, and Scenario 2 with M_C= 10,000 and h^2= 0.25. For computational efficiency, we used the aforementioned randomly sampled 50K unrelated British as the LD reference panel. We utilized the infinitesimal model implemented in LDpred (LDpred-inf) to calculate the posterior mean effects from GWAS summary statistics. For SBayesR, we used a sparse LD matrix (small LD correlation $|r|$ < 0.015 were set to 0) pre-calculated using the software GCTB[8] and ran all analyses with the default settings. We then created PGSs using the posterior mean effects estimated from LDpred-inf and SBayesR, respectively, and calculated the observed prediction R^2 and relative accuracies (RA) as described in the Methods section.

Supplementary Figures and Tables

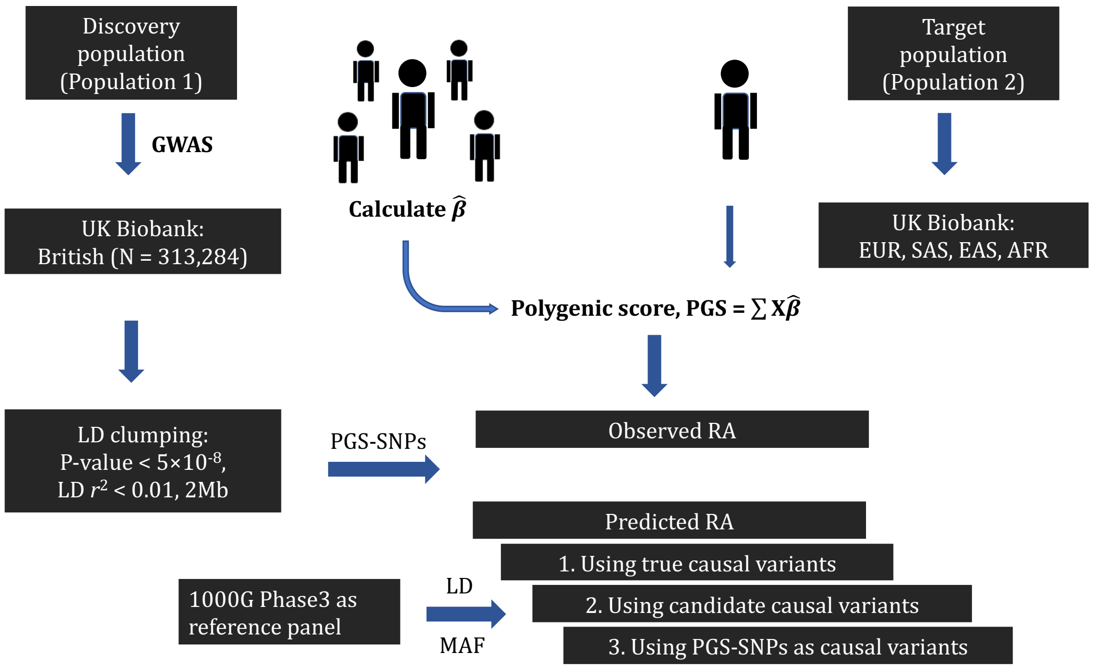

Supplementary Figure 1. Schematic overview of our study design. We ran GWASs in the discovery population (unrelated British with N=313,284) and then selected PGS-SNPs to generate PGSs using the LD clumping algorithm. We calculated both the empirical and deterministic relative accuracies in the target populations (including EUR, SAS, EAS and AFR in the UKB). We used three different approaches to calculate the deterministic relative accuracies (see the details in the main text).

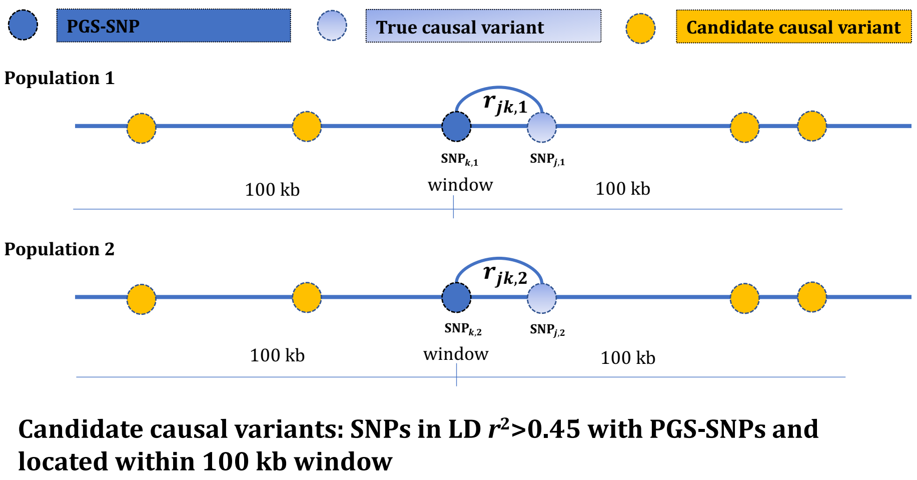

Supplementary Figure 2. The schematic example describing candidate causal variants. Noting the causal variants are generally unknown, we introduce the concept of candidate causal variants to approximate the LD correlation between PGS-SNP and causal variants ($r_{jk,l}$) in Population l.

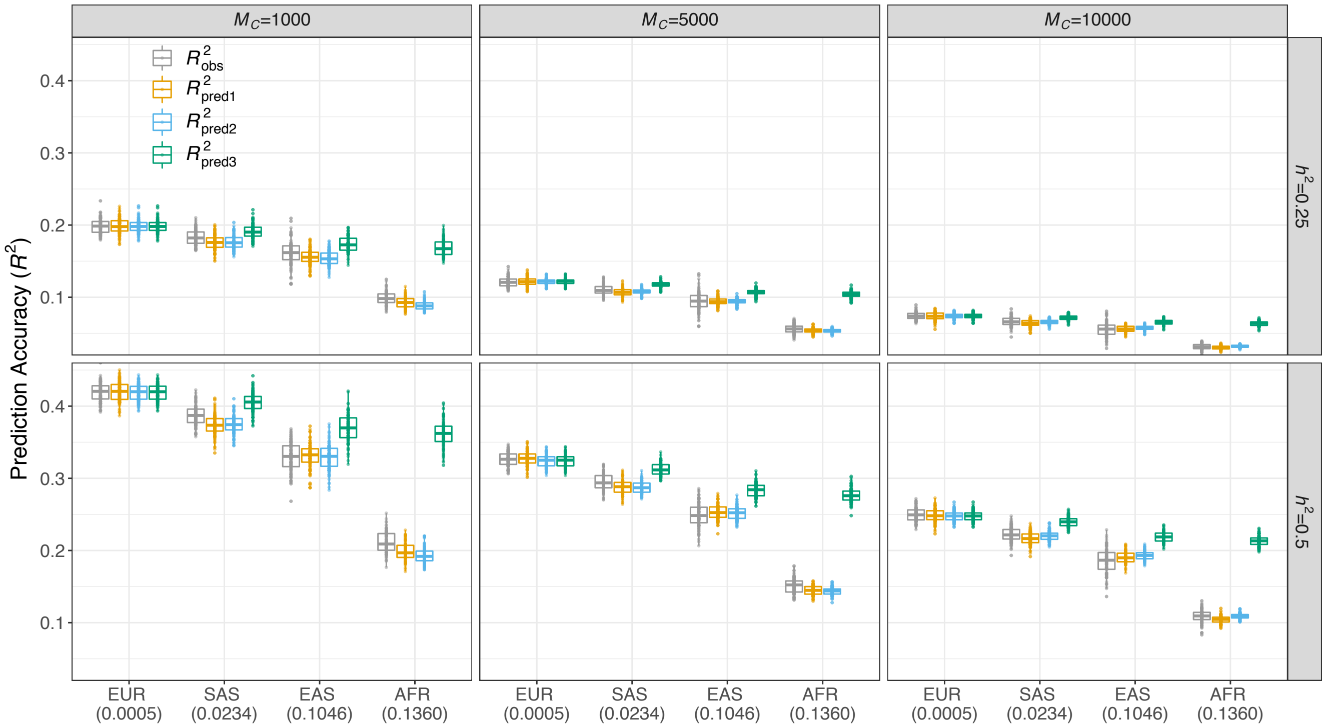

Supplementary Figure 3. Trans-ancestry prediction accuracy (R^2) of PGS in different simulation scenarios. We varied the trait heritability (h^2 = 0.25 and 0.5) and number of M_C causal variants (M_C = 1,000, 5,000 and 10,000) in the simulations. R^2_{obs} is the observed prediction R^2 in different ancestries. The predicted accuracy labelled as R^2_{pred1} is the R^2 estimated using Equation (1) in the main text based on parameters calculated from SNP pairs of PGS-SNPs and known causal variants within 100 kb; R^2_{pred2} is the R^2 calculated using SNP pairs of PGS-SNPs and candidate causal variants using Equation (2) in the main text; and R^2_{pred3} is referred to as the estimates of prediction R^2 using Equation (1) in the main text when assuming PGS-SNPs as causal variants. The numbers under the ancestry labels in x-axis denoted the pairwise F_{ST} calculated using HapMap 3 SNPs between discovery population and target population (see Supplementary Note 2). The boxes represent the first and third quantiles and whiskers are 1.5 folds the interquartile range. The points in each box are the estimates in 100 replicates. The median estimates are shown as the horizontal line in the boxes.

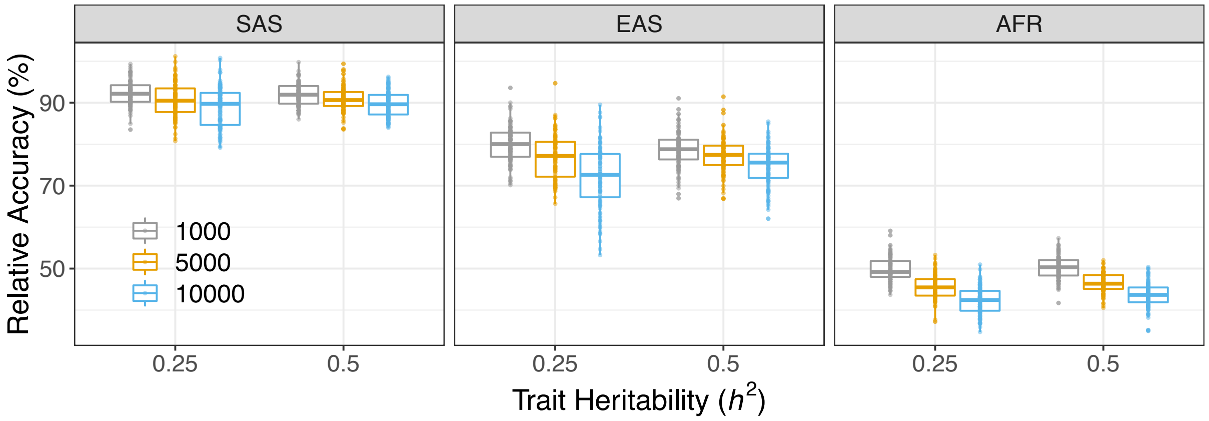

Supplementary Figure 4. The observed relative accuracies in different simulation scenarios. The observed relative accuracies (RA) were calculated as the ratio of the squared correlation between PGS and simulated trait in UKB participants of non-European ancestry over the same squared correlation estimated in 10,000 independent UKB participants of European ancestry. We varied the trait heritability (h^2 = 0.25 and 05) and number of M_C causal variants (M_C = 1,000, 5,000 and 10,000) in the simulations. We show that observed relative accuracies increase with the per SNP heritability (h^2/M_C), i.e. either decrease with increasing M_C or increase with increasing h^2. Boxes represent the first and third quantiles and whiskers are 1.5 folds the interquartile range. The points in each box are the estimates in 100 replicates. The median estimates are shown as the horizontal line in the boxes.

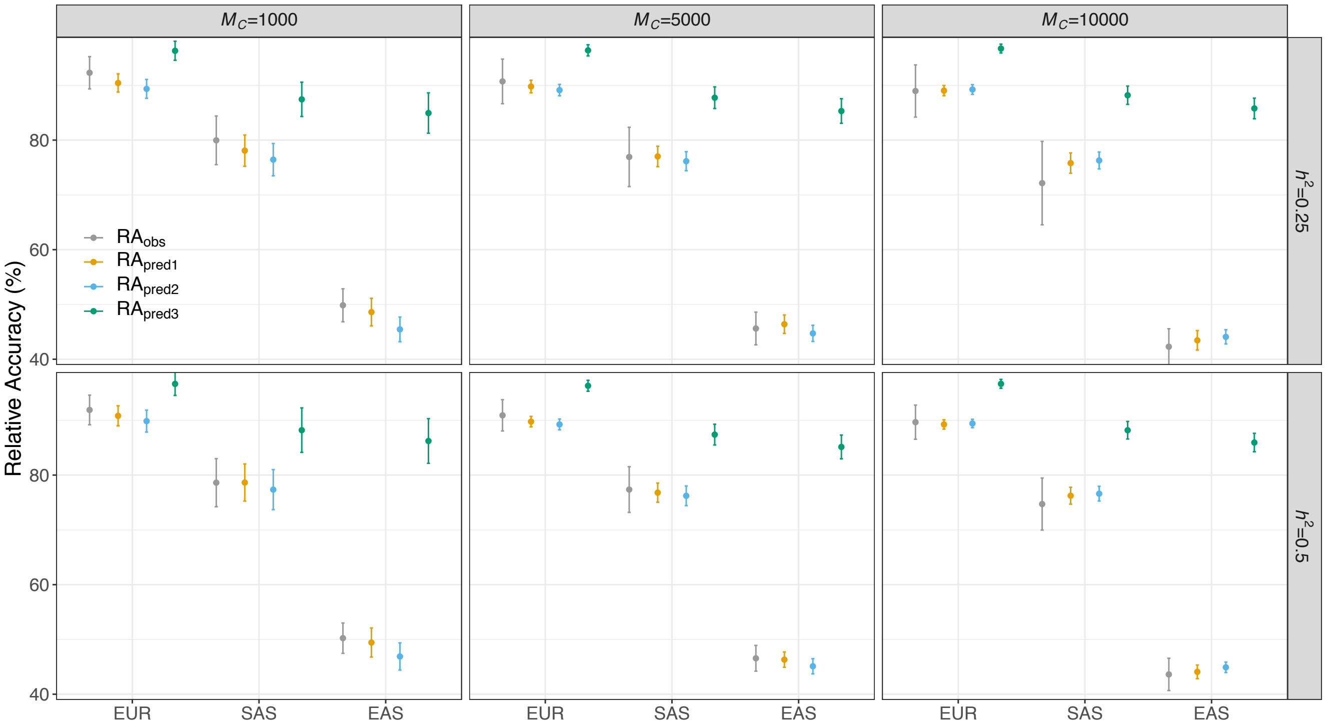

Supplementary Figure 5. Trans-ancestry relative accuracy of PGS in different simulation scenarios using the UKB imputed data as the reference panel. We varied the trait heritability (h^2 = 0.25 and 0.5) and number of M_C causal variants (M_C = 1,000, 5,000 and 10,000) in the simulations. The RA$_{obs}$, RA$_{pred1}$, RA$_{pred2}$ and RA$_{pred3}$ labels are the mean estimates which defined as the in the legend of Figure 1. We showed the results across scenarios were quite consistent with those obtained using 1KGP WGS data as the reference but with reduced underestimation (Figure 1). Error bars are standard deviations of the observed relative accuracy across 100 replicates.

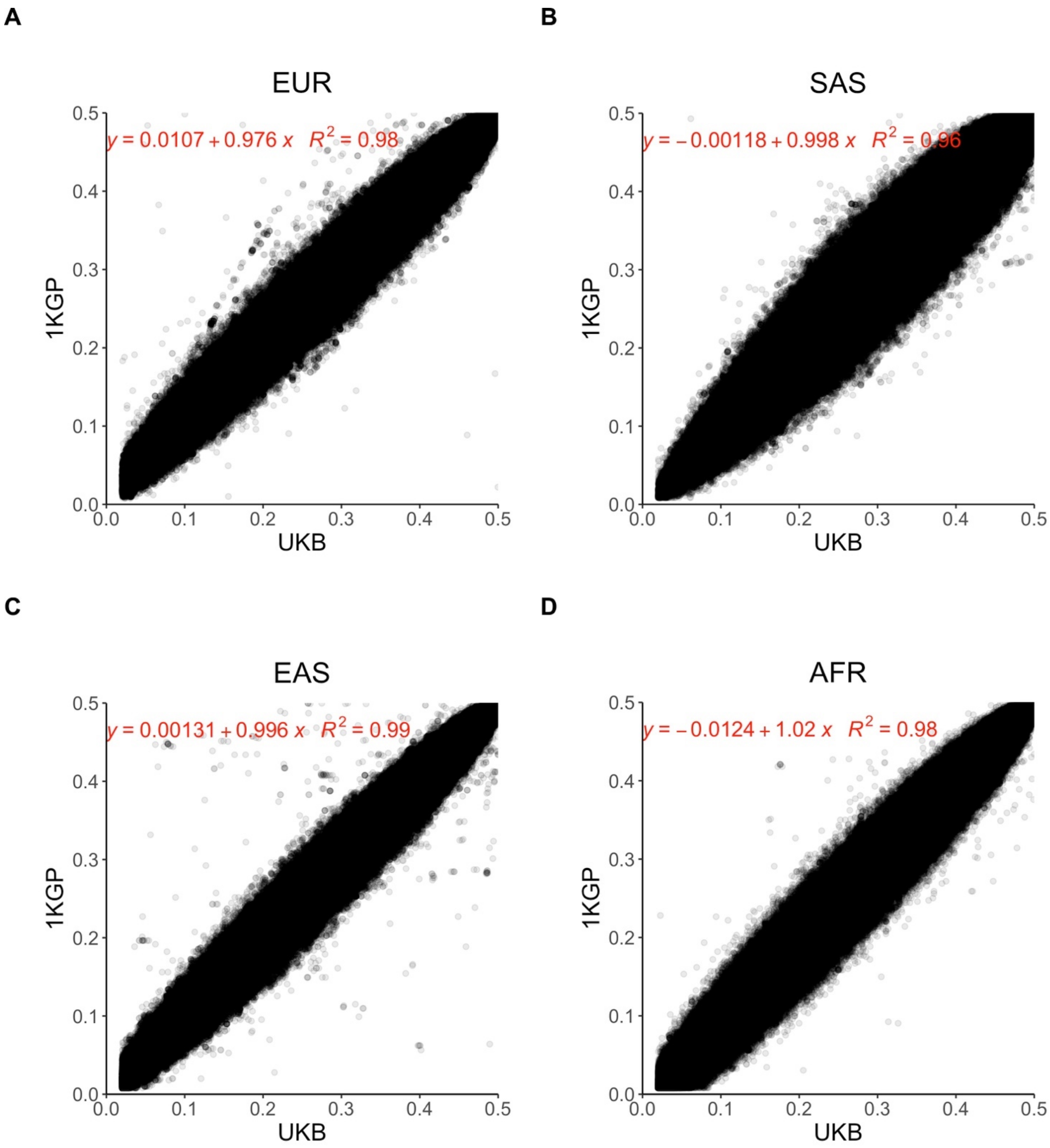

Supplementary Figure 6. Correlation of allele frequency between populations using different reference panels. We calculated the HapMap 3 SNP heterozygosity as $2p(1-p)$ in each ancestry from 1KGP WGS and UKB imputed data, respectively (see details in Supplementary Note 3).

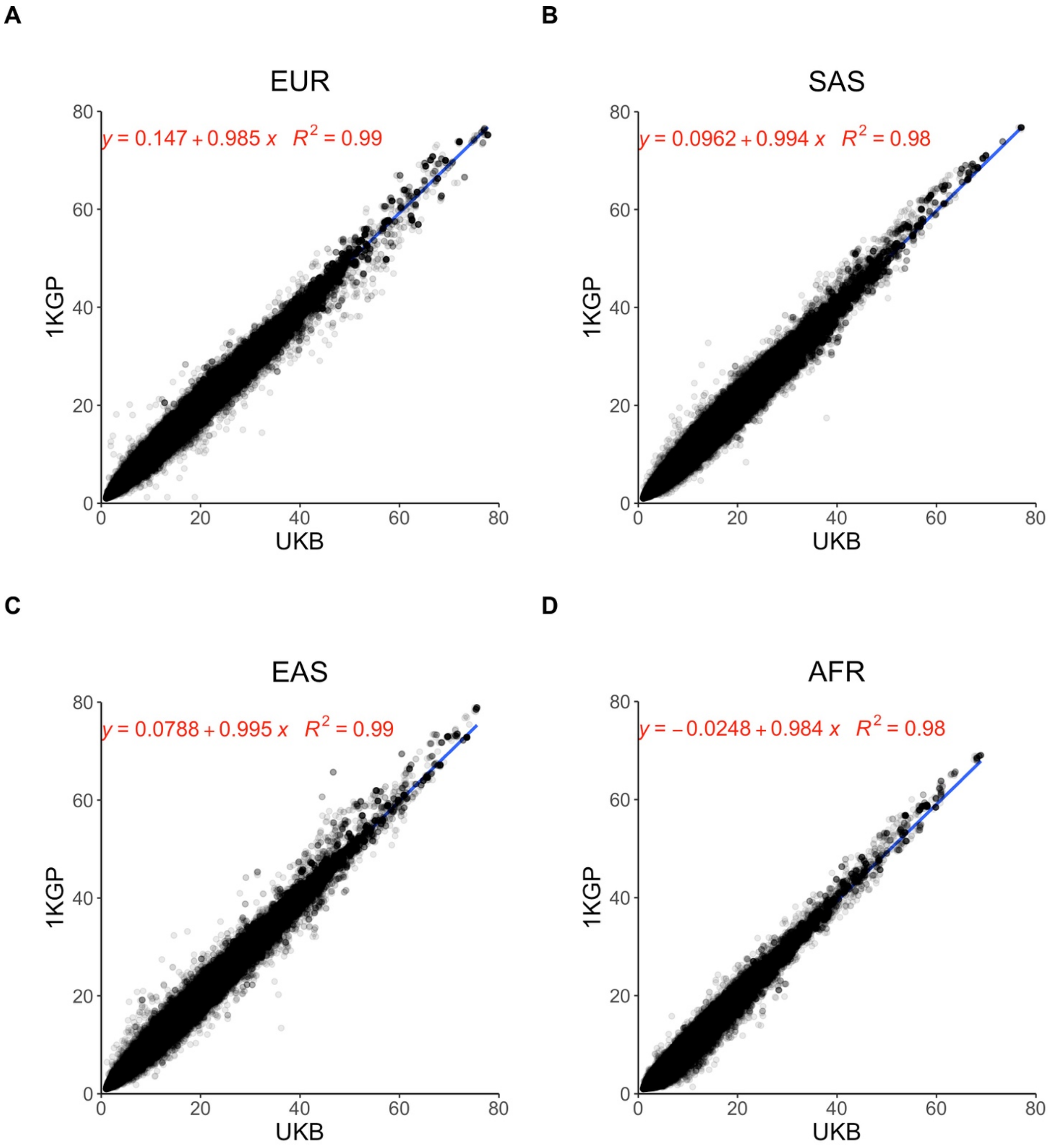

Supplementary Figure 7. Correlation of LD scores between populations using different reference panels. LD scores of HapMap3 SNPs were calculated within a 100 kb window in each ancestry from of the 1KGP (WGS data) and from the UKB (1KG-imputed data), respectively (see details in Supplementary Note 3).

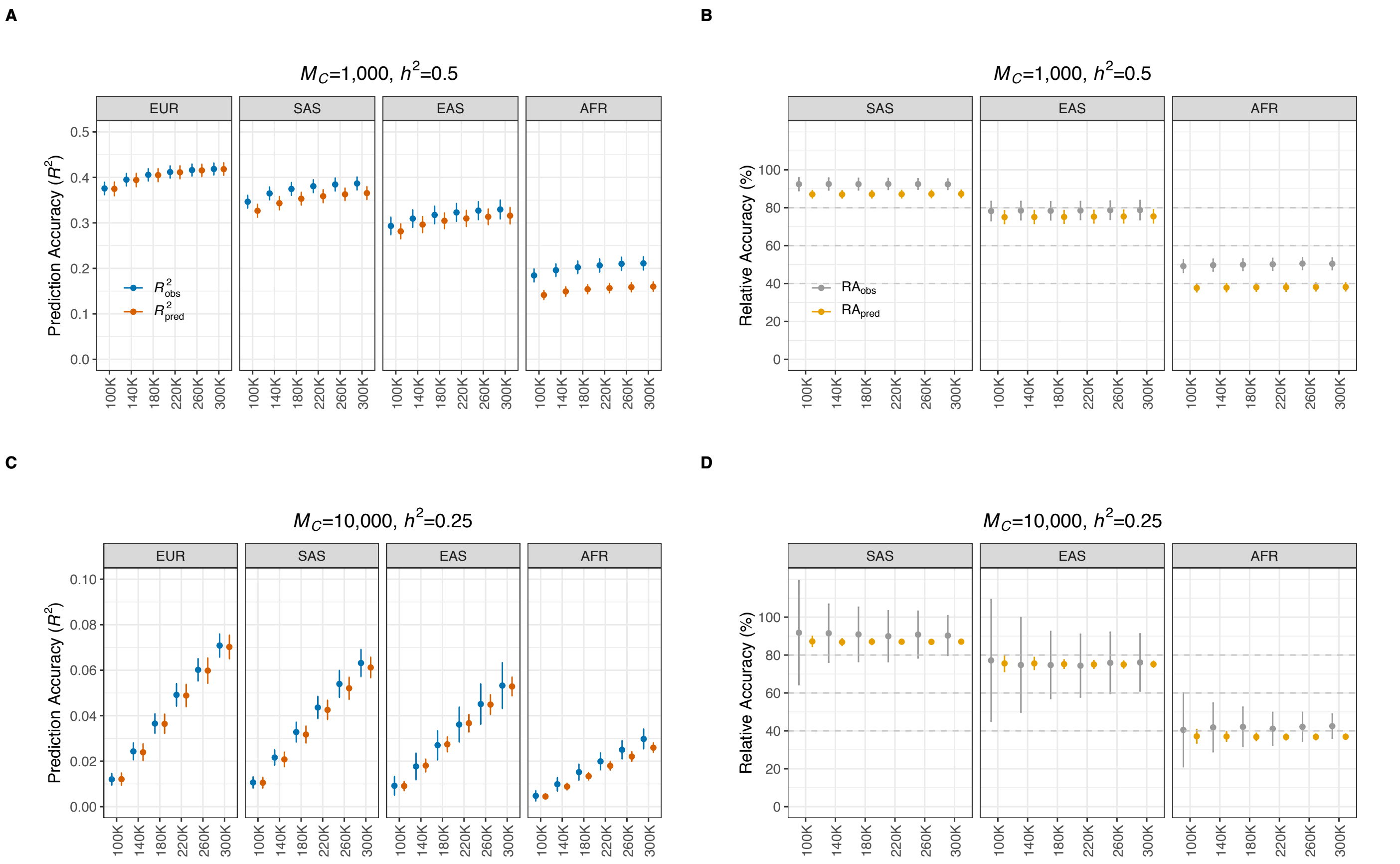

Supplementary Figure 8. Relative predictive performance of PGS in simulated data as a function of discovery GWAS sample sizes. We varied the GWAS sample sizes from 100,000 (100K) up to 300,000 (300K) (details in Supplementary Note 4) and quantified the impact of sample sizes on predictive performance in two different simulation scenarios. R^2_{obs} is the prediction R^2 calculated as the squared correlation between PGS and simulated trait in the target population; RA_{obs} is the observed RA; R^2_{pred} or RA_{pred} is the R^2 or RA calculated using SNP pairs of PGS-SNPs and candidate causal variants using Equation (2) in the main text. The dots are the mean estimates across 100 replicates. Error bars are standard deviations of the estimated prediction R^2 (A and C) or relative accuracy (B and D) across replicates. They grey dashed lines are y = 80%, y = 60% and y = 40%. The accuracy of PGS increases proportionally in all ancestries such that the RA of PGS from genome-wide significant SNPs is uncorrelated with sample size (Panels B and D).

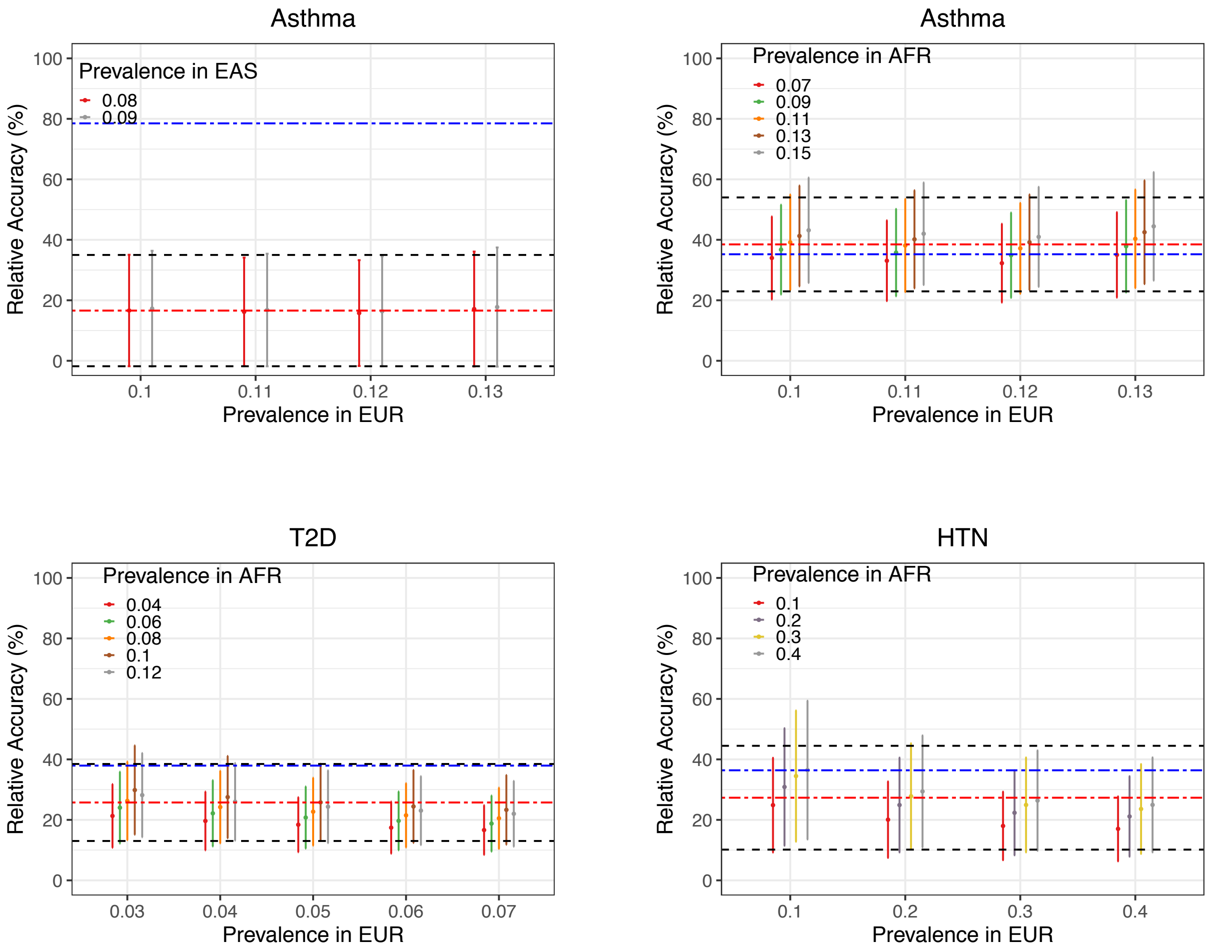

Supplementary Figure 9. Trans-ancestry relative prediction accuracy for diseases using ancestry specific prevalence from previous studies. We obtained ancestry specific prevalence for asthma[9,10], T2D[11–13] and HTN[13,14] and then ran sensitive analyses using those previously reported estimates in diseases with significant reduction of RA as shown in Fig. 3. The dashed lines are corresponding estimates using ancestry specific prevalence in the UKB. The red line is the observed RA, whilst the black lines are the standard errors. The blue line is RA predicted from Equation (2) in the main text only using information from LD and MAF differences between ancestries.

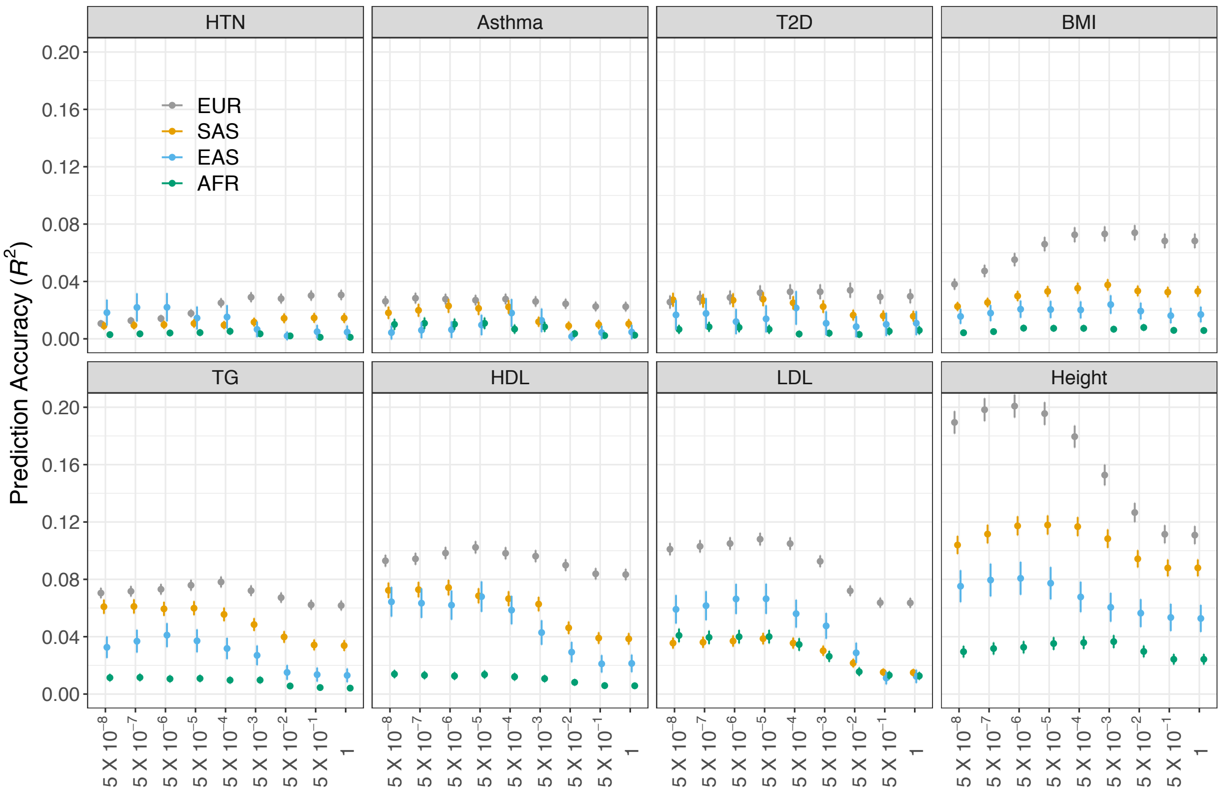

Supplementary Figure 10. Prediction accuracy at different *p*-value thresholds across traits and diseases in the UKB. We applied a range of *p*-value thresholds to select approximately independent SNPs using the algorithm LD clumping, details can be seen in Supplementary Note 6. The selected SNPs were then used to generate corresponding PGS to estimate the prediction accuracy. Error bars represent the standard errors of the prediction R^2 in each ancestry in the corresponding trait (see the derivation in Supplementary Note 7).

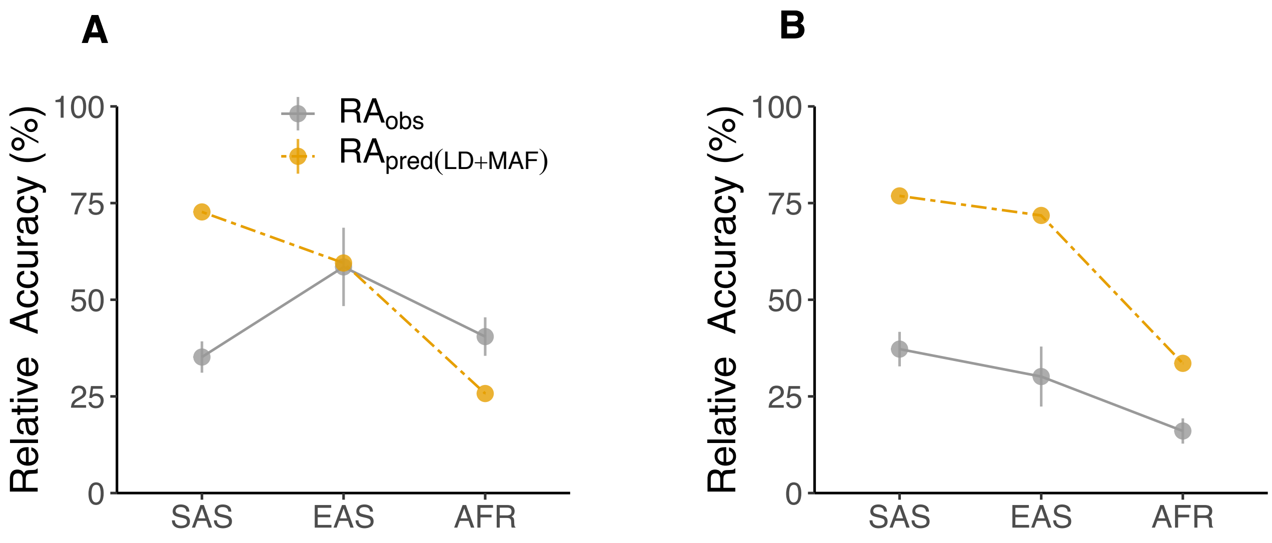

Supplementary Figure 11. Impact of inclusion of rs7254892 on relative accuracy of LDL PGS. A) The relative accuracy for LDL using all GWS SNPs. B) The relative accuracy for LDL after excluding the top GWS SNP (rs7254892). The RA_{obs} and $RA_{pred(LD+MAF)}$ labels are defined as the in legend of Fig. 3. Error bar in A and B represents the standard error for the observed RA for LDL PGS in each ancestry (see the derivation in Supplementary Note 7). The observed RA is reduced in EAS and AFR ancestries after excluding rs7254892.

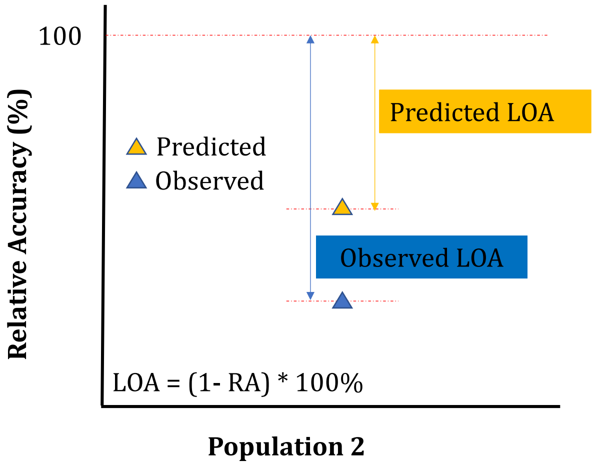

Supplementary Figure 12. Schematic description of the loss of accuracy (LOA). The ratio between the predicted LOA and observed LOA can be interpreted as the expected proportion of LOA attributable to the differences of LD and MAF between ancestries.

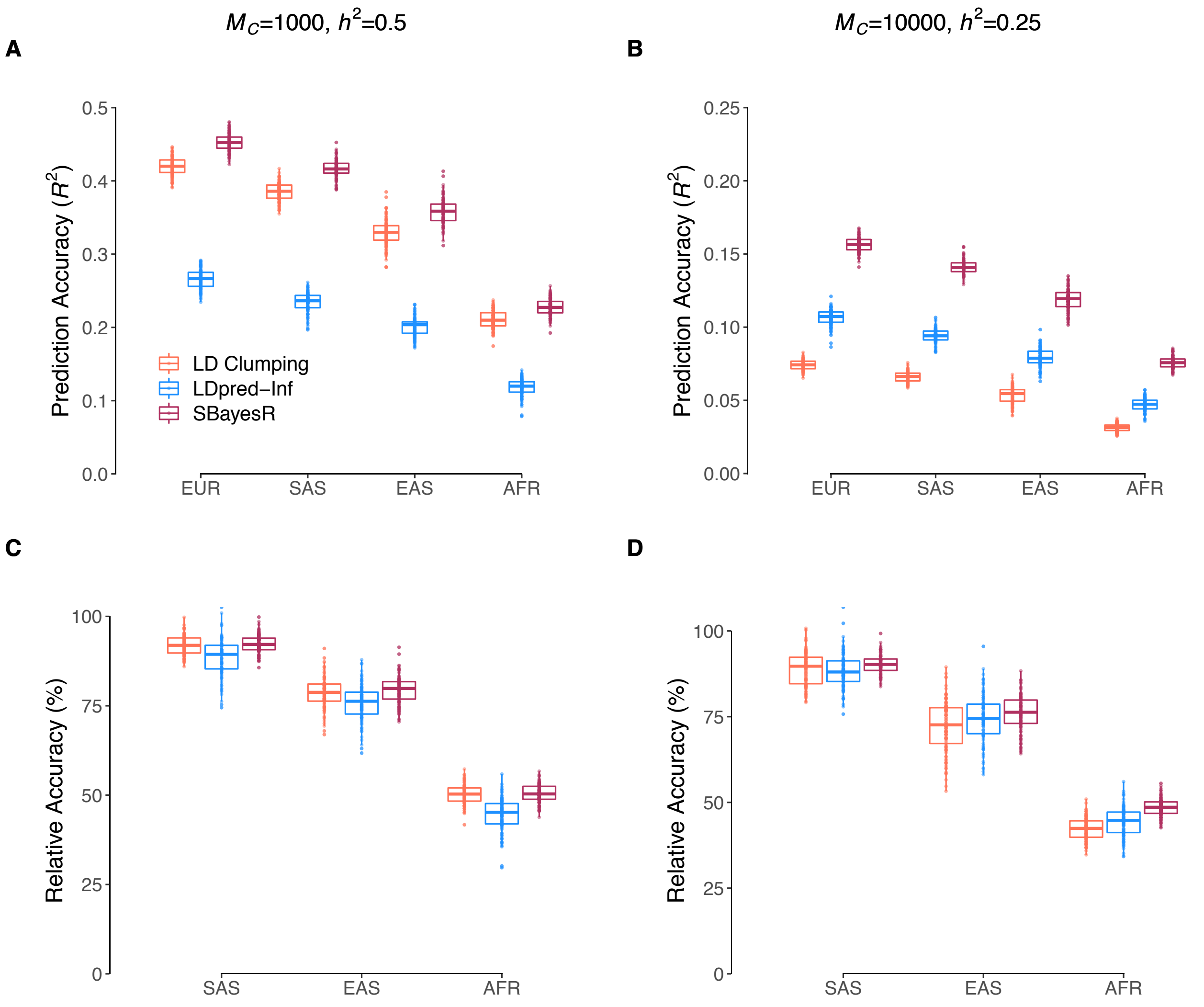

Supplementary Figure 13. Comparison of absolute and relative prediction accuracies of three polygenic scoring methods. We compared the prediction performance of polygenic scoring using LD clumping with two genome-wide methods, namely LDpred-inf and SBayesR, under two simulation scenarios. We find that SBayesR overperforms other methods in both scenarios in terms of the prediction R^2 whilst LDpred-inf showed improvement over LD clumping when M_C = 10,000 and h^2 = 0.25. As for the relative accuracy, SBayesR shows a slight improvement compared to other methods. Boxes represent the first and third quantiles and whiskers are 1.5 folds the interquartile range. The points in each box are the estimates in 100 simulation replicates. The median estimates are shown as the horizontal line in the boxes.

Supplementary Table 1. The number of GWS SNPs for traits/diseases in the UKB.

Trait	Abb.	GWS SNPs
Standing height	Height	1,182
Body mass index	BMI	338
LDL cholesterol	LDL	179
HDL cholesterol	HDL	271
Triglycerides	TG	178
Asthma	Asthma	71
Type 2 Diabetes	T2D	44
Hypertension	HTN	74

Supplementary Table 2. Trans-ancestry predictive performance of PGS of traits and diseases in the UKB across difference ancestries.

Trait	Ancestry	R^2_{obs} (S.E.)	$R^2_{pred(LD+MAF)}$	RA_{obs} (S.E.) %	$RA_{pred(LD+MAF)}$%	*p*-value	$\frac{(1-RA_{pred(LD+MAF)})}{(1-RA_{obs})}$ (S.E.) %
Height	EUR	0.193 (0.006)	0.194	100.0 (4.1)	100.0	1.0E+00	-
Height	SAS*	0.104 (0.006)	0.195	53.9 (3.6)	89.1	1.5E-37	23.6 (1.8)
Height	EAS*	0.075 (0.011)	0.184	39.0 (5.9)	76.7	4.7E-25	38.2 (3.7)
Height	AFR*	0.030 (0.004)	0.088	15.3 (2.1)	39.4	0.0E+00	71.5 (1.8)
BMI	EUR	0.035 (0.003)	0.035	100.0 (10.5)	100.0	1.0E+00	-
BMI	SAS*	0.023 (0.003)	0.031	64.1 (9.9)	87.6	2.9E-04	34.5 (9.5)
BMI	EAS*	0.016 (0.005)	0.028	44.4 (15.2)	77.3	2.5E-04	40.8 (11.2)
BMI	AFR*	0.004 (0.002)	0.014	12.0 (4.5)	38.6	3.7E-85	69.8 (3.6)
LDL	EUR	0.101 (0.004)	0.102	100.0 (6.0)	100.0	1.0E+00	-
LDL	SAS*	0.036 (0.004)	0.083	35.2 (4.1)	72.7	2.9E-56	42.1 (2.7)
LDL	EAS*	0.059 (0.010)	0.066	58.5 (10.1)	59.5	4.0E-05	97.6 (23.8)
LDL	AFR*	0.041 (0.005)	0.025	40.5 (5.0)	25.7	1.2E-32	124.9 (10.5)
HDL	EUR	0.093 (0.004)	0.093	100 (6.3)	100.0	1.0E+00	-
HDL	SAS*	0.072 (0.005)	0.085	77.8 (6.7)	89.3	9.2E-04	48.2 (14.5)
HDL	EAS*	0.064 (0.010)	0.071	69.2 (11.5)	74.0	7.4E-03	84.4 (31.5)
HDL	AFR*	0.014 (0.003)	0.038	15.0 (3.1)	35.8	1.6E-165	75.5 (2.8)
TG	EUR	0.070 (0.004)	0.071	100.0 (7.3)	100.0	1.0E+00	-
TG	SAS	0.061 (0.005)	0.060	86.5 (8.3)	86.5	1.0E-01	100.0 (61.5)
TG	EAS*	0.033 (0.007)	0.049	46.3 (10.9)	71.9	8.4E-07	52.3 (10.6)
TG	AFR*	0.011 (0.003)	0.027	16.2 (3.7)	36.5	1.4E-113	75.8 (3.3)
Asthma	EUR	0.026 (0.004)	0.026	100.0 (19.3)	100.0	1.0E+00	-
Asthma	SAS	0.018 (0.004)	0.022	69.4 (18.8)	85.1	1.0E-01	48.7 (29.9)
Asthma	EAS*	0.004 (0.005)	0.021	16.6 (18.4)	74.7	5.8E-06	30.3 (6.7)
Asthma	AFR*	0.010 (0.004)	0.009	38.5 (15.5)	34.0	7.3E-05	107.3 (27.0)
T2D	EUR	0.026 (0.005)	0.026	100.0 (25.2)	100.0	1.0E+00	-
T2D	SAS	0.027 (0.005)	0.022	106.1 (26.6)	88.4	8.2E-01	-190.2 (829.2)
T2D	EAS	0.017 (0.010)	0.023	65.0 (41.9)	88.8	4.0E-01	32.0 (38.3)
T2D	AFR*	0.007 (0.003)	0.010	25.7 (12.7)	39.2	4.9E-09	81.8 (14.0)

HTN	EUR	0.011 (0.002)	0.011	100.0 (27.9)	100.0	1.0E+00	-
HTN	SAS	0.009 (0.003)	0.009	85.2 (30.1)	90.8	6.2E-01	62.2 (126.4)
HTN	EAS	0.018 (0.009)	0.009	171.7 (90.7)	77.4	4.3E-01	-31.5 (39.9)
HTN	AFR*	0.003 (0.002)	0.004	27.3 (17.2)	37.7	2.4E-05	85.7 (20.3)

$R^2_{\mathrm{pred(LD+MAF)}}$ and $\mathrm{RA}_{\mathrm{pred(LD+MAF)}}$ refer to the prediction accuracy and RA, respectively, predicted from Equation (2) only using information from LD and MAF differences between ancestries. $\mathrm{RA}_{\mathrm{obs}}$ refers to the observed RA calculated using trait specific GWS SNPs. The proportion of the LOA explained by LD and MAF was calculated as $100\% \times (1-\mathrm{RA}_{\mathrm{pred(LD+MAF)}})/(1-\mathrm{RA}_{\mathrm{obs}})$. The standard error calculation for those estimates is derived in Supplementary Note 7. We calculated the *p*-values of reductions of $\mathrm{RA}_{\mathrm{obs}}$ using the Wald test. The asterisks indicate ancestry-trait/disease with a significant reduction in $\mathrm{RA}_{\mathrm{obs}}$ (p-value < 0.05) between EUR and non-EUR ancestries.

Supplementary References:

1. Weir, B. S. & Cockerham, C. C. Estimating F-Statistics for the Analysis of Population Structure. *Evolution (N. Y).* **38**, 1358–1370 (1984).
2. Chang, C. C. *et al.* Second-generation PLINK: Rising to the challenge of larger and richer datasets. *Gigascience* **4**, 7 (2015).
3. Yang, J., Lee, S. H., Goddard, M. E. & Visscher, P. M. GCTA: A tool for genome-wide complex trait analysis. *Am. J. Hum. Genet.* **88**, 76–82 (2011).
4. Zhu, Z. *et al.* Causal associations between risk factors and common diseases inferred from GWAS summary data. *Nat. Commun.* **9**, (2018).
5. Lynch, M. & Walsh., B. *Genetics and analysis of quantitative traits*. (Sunderland, MA: Sinauer, 1998).
6. Vilhjálmsson, B. J. *et al.* Modeling Linkage Disequilibrium Increases Accuracy of Polygenic Risk Scores. *Am. J. Hum. Genet.* **97**, 576–592 (2015).
7. Lloyd-Jones, L. R. *et al.* Improved polygenic prediction by Bayesian multiple regression on summary statistics. *Nat. Commun.* **10**, 522961 (2019).
8. Zeng, J. *et al.* Signatures of negative selection in the genetic architecture of human complex traits. *Nat. Genet.* **50**, 746–753 (2018).
9. To, T. *et al.* Global asthma prevalence in adults: Findings from the cross-sectional world health survey. *BMC Public Health* **12**, (2012).
10. The Global Asthma Report Asthma affects. (2018). Available at: http://www.globalasthmareport.org/Global Asthma Report 2018.pdf.
11. Ntuk, U. E., Gill, J. M. R., Mackay, D. F., Sattar, N. & Pell, J. P. Ethnic-specific obesity cutoffs for diabetes risk: Cross-sectional study of 490,288 uk biobank participants. *Diabetes Care* **37**, 2500–2507 (2014).
12. Saeedi, P. *et al.* Global and regional diabetes prevalence estimates for 2019 and projections for 2030 and 2045: Results from the International Diabetes Federation Diabetes Atlas, 9th edition. *Diabetes Res. Clin. Pract.* **157**, (2019).
13. Schofield, P., Saka, O. & Ashworth, M. Ethnic differences in blood pressure monitoring and control in South East London. *Br. J. Gen. Pract.* **61**, (2011).
14. Mills, K. T. *et al.* Global disparities of hypertension prevalence and control. *Circulation* **134**, 441–450 (2016).
