# Supplementary material for: Theoretical and empirical quantification of the accuracy of polygenic scores in ancestry divergent populations
Source: Nat Commun. 2020 Jul 31;11:3865. doi: 10.1038/s41467-020-17719-y (PMC7395791; doi:10.1038/s41467-020-17719-y)
Supplement: Supplementary file 3 — Reporting Summary [file 41467_2020_17719_MOESM3_ESM.pdf]

nature research

Corresponding author(s): Dr Loic Yengo

Last updated by author(s): Jul 6, 2020

Reporting Summary

Nature Research wishes to improve the reproducibility of the work that we publish. This form provides structure for consistency and transparency in reporting. For further information on Nature Research policies, see Authors & Referees and the Editorial Policy Checklist .

Statistics

For all statistical analyses, confirm that the following items are present in the figure legend, table legend, main text, or Methods section.

n/a	Confirmed	
☐	☒	The exact sample size (*n*) for each experimental group/condition, given as a discrete number and unit of measurement
☒	☐	A statement on whether measurements were taken from distinct samples or whether the same sample was measured repeatedly
☐	☒	The statistical test(s) used AND whether they are one- or two-sided<br>*Only common tests should be described solely by name; describe more complex techniques in the Methods section.*
☐	☒	A description of all covariates tested
☐	☒	A description of any assumptions or corrections, such as tests of normality and adjustment for multiple comparisons
☐	☒	A full description of the statistical parameters including central tendency (e.g. means) or other basic estimates (e.g. regression coefficient) AND variation (e.g. standard deviation) or associated estimates of uncertainty (e.g. confidence intervals)
☐	☒	For null hypothesis testing, the test statistic (e.g. *F*, *t*, *r*) with confidence intervals, effect sizes, degrees of freedom and *P* value noted<br>*Give P values as exact values whenever suitable.*
☒	☐	For Bayesian analysis, information on the choice of priors and Markov chain Monte Carlo settings
☒	☐	For hierarchical and complex designs, identification of the appropriate level for tests and full reporting of outcomes
☐	☒	Estimates of effect sizes (e.g. Cohen's *d*, Pearson's *r*), indicating how they were calculated

Our web collection on statistics for biologists contains articles on many of the points above.

Software and code

Policy information about availability of computer code

Data collection	No software were used for data collection.
Data analysis	Statistical analyses were performed mostly with R version v3.2 and v3.5. Other analyses were performed using PLINK v1.9.

For manuscripts utilizing custom algorithms or software that are central to the research but not yet described in published literature, software must be made available to editors/reviewers. We strongly encourage code deposition in a community repository (e.g. GitHub). See the Nature Research guidelines for submitting code & software for further information.

Data

Policy information about availability of data

All manuscripts must include a data availability statement. This statement should provide the following information, where applicable:
- Accession codes, unique identifiers, or web links for publicly available datasets
- A list of figures that have associated raw data
- A description of any restrictions on data availability

We used genotypic data and phenotypic data from the UK biobank under project number 12505. UKB data can be accessed upon request once a research project has been submitted and approved by the UKB reserach committee. We also used publicly available genotypes data from 2504 participants of the 1000 genomes project.

Field-specific reporting

Please select the one below that is the best fit for your research. If you are not sure, read the appropriate sections before making your selection.

Life sciences study design

All studies must disclose on these points even when the disclosure is negative.

Sample size	We used pre-existing data from the UK Biobank (N=487,371).
Data exclusions	We used all data made available.
Replication	We provide source codes for reproducing the different simulations results and data analyses presented in the manuscript.
Randomization	N/A
Blinding	N/A

Reporting for specific materials, systems and methods

We require information from authors about some types of materials, experimental systems and methods used in many studies. Here, indicate whether each material, system or method listed is relevant to your study. If you are not sure if a list item applies to your research, read the appropriate section before selecting a response.

Materials & experimental systems

n/a	Involved in the study	
☒	☐	Antibodies
☒	☐	Eukaryotic cell lines
☒	☐	Palaeontology
☒	☐	Animals and other organisms
☐	☒	Human research participants
☒	☐	Clinical data

Methods

n/a	Involved in the study	
☒	☐	ChIP-seq
☒	☐	Flow cytometry
☒	☐	MRI-based neuroimaging

Human research participants

Policy information about studies involving human research participants

Population characteristics	For our GWAS of height, BMI, LDL and HDL cholesterol, triglycerides, type 2 diabetes, hypertension and asthma, analyses were adjusted for age at recruitment, recruitment centre (treated as a categorical factor), sex, year of birth (treated as a continuous variable), genotyping batch (treated as a factor), and population structure measured by 10 genetic principal components.
Recruitment	UK Biobank investigators sent postal invitations to 9,238,453 individuals registered with the UK's National Health Service who were aged 40–69 years and lived within approximately 25 miles (40 km) of one of 22 assessment centers located throughout England, Wales, and Scotland. Overall, 503,317 participants consented to join the study cohort and visited an assessment center between 2006 and 2010, resulting in a participation rate of 5.45%. (Fry et al. Am J Epidemiol. 2017 Nov 1;186(9):1026-1034).
Ethics oversight	The National Information Governance Board for Health and Social Care and the North West Multicentre Research Ethics Committee provided approval for UK Biobank to obtain the contact details of people within the eligible age range from local National Health Service Primary Care Trusts.

Note that full information on the approval of the study protocol must also be provided in the manuscript.
